# The Use of Point-of-Care Tests and Multiplex PCR Tests in the Pediatric Emergency Department Reduces Antibiotic Prescription in Patients with Febrile Acute Respiratory Infections

**DOI:** 10.3390/pathogens14121284

**Published:** 2025-12-13

**Authors:** Luca Pierantoni, Arianna Dondi, Liliana Gabrielli, Valentina Lasala, Laura Andreozzi, Laura Bruni, Fiorentina Guida, Eleonora Battelli, Giulia Piccirilli, Ilaria Corsini, Tiziana Lazzarotto, Marcello Lanari, Daniele Zama

**Affiliations:** 1 Pediatric Unit, IRCCS Azienda Ospedaliero-Universitaria of Bologna, 40138 Bologna, Italy; luca.pierantoni@aosp.bo.it (L.P.); arianna.dondi@aosp.bo.it (A.D.); laura.andreozzi@aosp.bo.it (L.A.); fiorentina.guida@unibo.it (F.G.); eleonora.battelli@aosp.bo.it (E.B.); ilaria.corsini2@unibo.it (I.C.); marcello.lanari@unibo.it (M.L.); daniele.zama2@unibo.it (D.Z.); 2 Microbiology Unit, IRCCS Azienda Ospedaliero-Universitaria di Bologna, 40138 Bologna, Italy; liliana.gabrielli@aosp.bo.it (L.G.); giulia.piccirilli@aosp.bo.it (G.P.); tiziana.lazzarotto@unibo.it (T.L.); 3 Speciality School of Paediatrics, Alma Mater Studiorum, University of Bologna, 40138 Bologna, Italy; laurabrni@yahoo.it; 4 Department of Medical and Surgical Sciences, Alma Mater Studiorum, University of Bologna, 40138 Bologna, Italy

**Keywords:** acute respiratory infection, pediatric emergency department, antibiotic prescription, point-of-care tests, antimicrobial stewardship programs

## Abstract

Background: Acute Respiratory Infections are a common reason for Pediatric Emergency Department (PED) visits. Differentiating bacterial and viral infections may be challenging and might result in incorrect antibiotic prescriptions and exacerbation of antimicrobial resistance. This study evaluated the impact of new diagnostic tests in PED. Methods: A retrospective cohort of 4882 acute febrile respiratory infection cases presenting to the PED was analyzed, comparing two periods: Period 1 (October 2016–March 2017, *n* = 2181) and Period 2 (October 2023–March 2024, *n* = 2701). During Period 1, Group A Streptococcus and Respiratory Syncytial Virus rapid antigen detection tests were available. During Period 2, new point-of-care tests (POCTs), including rapid C-reactive protein and rapid antigen detection for Influenza A, Influenza B, and SARS-CoV-2, and a multiplex PCR nasal swab, were introduced. Results: In Period 2, antibiotic prescriptions decreased by 28.4%, along with a reduction in broad-spectrum antibiotic use. A significant correlation was observed between reduced antibiotic prescription and the use of new POCTs and multiplex PCR tests. Performance of blood tests and chest radiographs also decreased. Conclusions: Implementing novel diagnostic tests in PED helps clinicians select more appropriate management options with an impact on reduced stress and radiation exposure and antibiotic prescription.

## 1. Introduction

Fever and acute respiratory infections (ARIs) are the most common reasons for attending a paediatric emergency department (PED). In particular, upper respiratory tract infections (URTIs), which are generally mild and self-limiting, account for about 50% of fever-related evaluations in PED [1,2]. Most cases of ARIs require only supportive therapy, as the aetiology is viral [3,4,5,6]. Timely recognition of bacterial infections is essential to initiate appropriate antibiotic therapy. However, differentiating bacterial from viral infections based solely on clinical presentation remains challenging due to the considerable overlap of signs and symptoms. In the context of respiratory infections with suspected or confirmed bacterial aetiology, there is a consensus among guidelines on the use of narrow-spectrum antibiotics, such as amoxicillin, as the first-line treatment option. Broad-spectrum antibiotics are reserved for complicated cases, unvaccinated children, or in the event of allergies [7,8]

Antibiotics are among the most frequently prescribed medications in paediatrics. Nevertheless, a substantial proportion of these prescriptions has been reported to be unnecessary or inappropriate. The multicenter study “MOFICHE”, which evaluated uncomplicated cases of respiratory infections presenting at various European PEDs, highlighted that 42% of antibiotic prescriptions were inappropriate in terms of duration, indications, and/or adherence to guidelines [2].

The inappropriate use of antibiotic therapy has contributed to the escalating prevalence of antimicrobial resistance and may be associated with avoidable adverse events [9,10]. In recent years, major global organizations have expressed concern over the rising rates of antimicrobial resistance and have urged the adoption of programs to limit the spread of multidrug-resistant organisms. These programs, known as antimicrobial stewardship programs [11,12,13], should be proactive and aimed at optimizing antibiotic prescribing practices. Diagnostic stewardship was designed to enhance the appropriate utilization of diagnostic tests across different clinical settings, thereby ensuring optimal patient management and treatment [11,13,14,15]. Moreover, clinical uncertainty contributes to prolonged waiting times in the PED, heightens the likelihood of hospital admission, and increases the financial burden on patients. The decision-making process can be further influenced by external pressures, including those exerted on healthcare providers and parents [16].

Point-of-care tests (POCTs) are diagnostic procedures that are performed in proximity to the patient, with the notable advantage of providing rapid results [11,17]. Their implementation has been suggested to help reduce inappropriate antibiotic prescriptions [11,15,17], a shorter duration of antibiotic therapy [18], and decreased hospital bed utilization, potentially resulting in cost savings [11,17,19,20]. Current scientific literature is inconclusive and presents conflicting data on the real impact of POCTs on PED. It remains unclear whether the availability of such methods provides real advantages in selecting the most appropriate therapy and in reducing the need for additional diagnostic tests [11,15,21,22,23].

This study aims to evaluate the impact of new diagnostic tools on the management of pediatric patients with febrile ARIs attending PED, specifically assessing whether the introduction of these new diagnostic tools contributed to a reduction in antibiotic prescriptions and diagnostic examinations.

## 2. Materials and Methods

### 2.1. Study Design

This is a monocentric, observational, retrospective cohort study that included patients aged between 0 and 14 years who were referred to the PED at the IRCCS Azienda Ospedaliero-Universitaria in Bologna (Italy), Policlinico of Sant’Orsola, a tertiary-level, university-affiliated hospital, which manages approximately 25,000 visits per year.

The inclusion criteria comprised the following: (1) age between 0 and 14 years; (2) body temperature > 37 °C; (3) diagnosis of ARIs at discharge: URTIs (including rhinitis, tonsillitis and pharyngitis) croup, bronchiolitis, bronchitis, and pneumonia. Patients with SARS-CoV-2 infection were excluded, whereas patients with comorbidities were included.

The study population was divided into two distinct periods, defined as before or after the implementation of new diagnostic tools. In Period 1 (October 2016–March 2017), the available POCT methods included only the Group A Streptococcus rapid antigen detection (GAS-RAD) test and the Respiratory Syncytial Virus rapid antigenic detection (RSV-RAD) test. In addition, the Virology Laboratory had direct immunofluorescence assays (DFAs) available for Respiratory Syncytial Virus, Influenza A and Influenza B viruses, Parainfluenza viruses 1–3, Metapneumovirus, and Adenovirus, which could be used, although it was not systematically used, mainly for logistic reasons.

Period 2 (October 2023–March 2024) incorporates novel diagnostic instruments, including C-Reactive Protein Point-of-Care (CRP-POC) test, a rapid multiple antigen test for SARS-CoV-2, Influenza A and Influenza B (COMBO test) and a multiplex Polymerase Chain Reaction (PCR) test for Respiratory Syncytial virus, Influenza A and Influenza B viruses, Parainfluenza viruses 1–4, Metapneumovirus, Adenovirus, and Rhinovirus.

The primary objective of this study was to compare the impact of POCTs and multiplex PCR tests implementation on antibiotic prescriptions for febrile pediatric patients with ARIs admitted to the PED between Period 1 and Period 2. The secondary objectives were to assess differences in the utilization of diagnostic tests, length of stay (LOS) in the PED, the number of hospitalizations, and admissions to short-stay observation (SSO) between the two periods. Additionally, the study aimed to identify variables associated with antibiotic prescribing.

### 2.2. Data Collection

Study personnel reviewed electronic medical records to collect demographic and clinical data. These included age, gender, weight, heart rate, body temperature, peripheral oxygen saturation, presence of body temperature ≥ 38 °C, fever duration in days, presence and type of antibiotic therapy at the time of admission to the PED, and presence and type of comorbidity at the time of admission to the PED. The study staff also reviewed the investigations performed in the PED, including laboratory blood tests, chest X-rays (CXRs), GAS-RAD tests, RSV-RAD tests, COMBO tests, multiplex PCR tests, and CRP-POC tests. Furthermore, the outcomes of the patients’ visits, such as discharge diagnosis, antibiotic therapy prescribed at discharge, hospitalizations or admissions to SSO, and LOS at the PED, were thoroughly assessed. Regarding the antibiotic prescription, data on the type of molecule used and the length of therapy were also collected.

### 2.3. Rapid and Microbiological Diagnostic Test

In both Period 1 and Period 2, GAS-RAD and RSV-RAD tests were available. The former immunochromatographic test was conducted using the OneCheck STREPTO-A kit (DeltaChemie Biotechnology, Naples, Italy) through the collection of a throat swab specimen, with results available within 5 min. The latter is a rapid immunochromatographic immunoassay for the detection of RSV antigens in nasal wash samples, which was performed by medical staff during the clinical visit using the tru-RSV kit (Meridian Bioscience, Newtown, OH, USA), with results available within 15 min.

Before 2022 in the Virology Laboratory on nasopharyngeal aspirates, antigens from eight respiratory viruses were detected using DFAs: Respiratory Syncytial virus, Influenza A and Influenza B viruses, Parainfluenza viruses 1–3, and Adenovirus were identified using the SimulFluor^®^ Respiratory Screening Kit (Light Diagnostics™ Chemicon International, Temecula, CA, USA), while Human Metapneumovirus was detected using a Human Metapneumovirus direct immunofluorescence assay (Light Diagnostics™).

In Period 2, three additional tests were introduced: the CRP-POC, the COMBO test, and direct immunofluorescence assays were replaced by a multiplex PCR test, which was also validated for use on nasopharyngeal swabs (Table 1).

The CRP-POC was carried out using the CRP kit from EUROLyser Diagnostica GmbH (Salzburg, Austria), using a finger-prick, with results available within 5 min. The COMBO test is performed on a nasopharyngeal swab to detect antigens of SARS-CoV-2, Influenza A, and Influenza B. It was carried out using the AFIAS-6 COVID-19/Flu A + B Combo kits (BioTechnology Boditech, Chuncheon-si, Republic of Korea), with results available within 20 min. In PED in the months following Period 2, the detection of Respiratory Syncytial virus antigens was also added to the COMBO test, using the AFIAS-6 COVID-19/Flu A + B/VRS Combo kits (BioTechnology Boditech).

Regarding the multiplex PCR test, biological samples were collected via nasopharyngeal swab and sent to the Microbiology Unit for PCR testing, performed using the Allplex™ RV Essential Assay kit (Seegene, Seoul, Republic of Korea). Sample processing occurred within 6 h, and results were available within 24 h. This system enables the simultaneous detection of the genomes of Respiratory Syncytial virus, Influenza A and Influenza B viruses, Parainfluenza types 1–4 viruses, Human Metapneumovirus, Adenovirus, and Rhinovirus.

PED pediatricians received technical training on sample collection and processing, but the decision to perform the test, as well as the choice of which test to use, and the interpretation of the result, were at the discretion of the pediatrician.

### 2.4. Statistical Analysis

The statistical analysis was conducted using R version 4.4.2 (31 October 2024). In view of the large sample size, the normality of the analyzed variables was ascertained through the utilization of visual inspection methods, including histograms and Q-Q plots. Categorical variables were reported as absolute frequencies and percentages, whereas continuous variables were presented as mean and standard deviation (SD) or median and interquartile range (IQR), depending on whether their distribution was normal or non-normal, respectively. Patients were divided into two groups based on the period of admission to the PED: before or after the implementation of point-of-care tests.

The differences between categorical variables in the group data were analyzed using the chi-square test, and continuous variables were compared using either Student’s *t*-test or Mann–Whitney U test, as appropriate. A detailed investigation was conducted on standardized residuals, specifically in instances where the non-binomial chi-square test demonstrated statistical significance.

Missing data were handled through listwise deletion, given the low proportion of missing values.

The effect sizes were reported for all primary group comparisons, using Cohen’s d for *t*-tests, rank-biserial correlation for Mann–Whitney U tests, and Cramer’s V for chi-square analyses. Cohen’s d effect sizes of 0.2, 0.5, and 0.8 were interpreted as representing small, medium, and large effects for comparisons, respectively. Effect sizes were interpreted based on the following thresholds: values below 0.1 were considered negligible or likely to represent random variation; values between 0.1 and 0.3 were regarded as small, potentially relevant in large samples or for sensitive outcomes; values between 0.3 and 0.5 were interpreted as moderate and likely to have clinical relevance; values between 0.5 and 0.7 were considered moderately strong, indicating a consistent and clinically useful effect; and values greater than 0.7 were interpreted as large, reflecting a strong and potentially impactful association. These thresholds were defined a priori based on expected clinical relevance and empirical considerations within pediatric research contexts. The effect size measure calculated for Chi^2^-tests was Cramer’s V (>0.1 = small; >0.3 = medium; >0.5 = large effect [24].

Univariate and multivariate logistic regression analyses were performed to assess the determinants of systemic antibiotic prescription in the study population. Variables with *p* < 0.20 in unadjusted analyses were considered for inclusion in the multivariable model. The independent variables included in unadjusted analyses were period of admission to the PED, LOS in PED, age in months, gender, days of fever, body temperature ≥ 38 °C, presence of comorbidities, performance of blood tests, performance of CXRs, performance of POCTs, performance of multiplex PCR test, and discharge diagnosis. The reference category for the variable “diagnosis” was set to “pneumonia” in the logistic regression model. Collinearity was assessed using the variance inflation factor, which remained consistently below 2. Odds ratios and 95% confidence intervals were calculated, and a *p*-value < 0.05 was considered statistically significant.

### 2.5. Ethical Approval

The study was approved by the Ethics Committee of the Emilia Centro Area Vasta (CE-AVEC), protocol DIARIO, with registration number 229/2025/Oss/AOUBo.

## 3. Results

During the two epidemic seasons, a total of 4882 patients presenting in PED with febrile ARIs were enrolled: 2181 patients in Period 1 and 2701 in Period 2.

The clinical and demographic characteristics of the study cohort are summarized in Table 2. Over the entire study period, 793 (16.2%) subjects were already receiving antibiotic therapy at the time of admission to the PED, with no statistically significant difference between the two periods. Additionally, 225 (10.3%) patients in Period 1 and 341 (12.6%) patients in Period 2 had comorbidities (*p* = 0.014). In the post hoc analysis, lung disease comorbidity mainly contributed to the statistically significant difference between the two periods (*p* = 0.032).

Regarding diagnosis, the post hoc analysis of diagnoses at discharge showed an increase in diagnoses of URTI during Period 2 (*p* < 0.001) and of croup and bronchitis during Period 1 (*p* = 0.003 for both). No statistically significant differences were observed for bronchiolitis and pneumonia.

**Figure 1:**
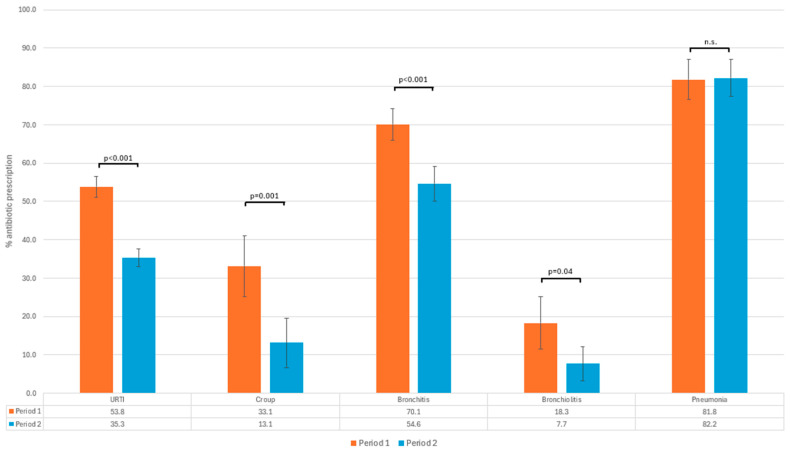
Post hoc analysis about differences in antibiotic prescription according to diagnosis of discharge (URTI, croup, bronchitis, bronchiolitis, and pneumonia) in Period 1 and Period 2. Error bars representing 95% confidence intervals and *p*-values were included. URTI = upper respiratory tract infection.

**Figure 2:**
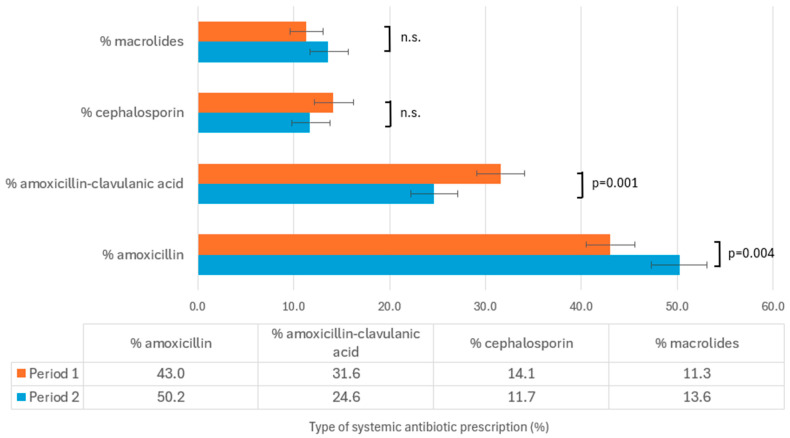
Post hoc analysis about differences in the type of antibiotic prescription (amoxicillin, amoxicillin-clavulanic acid, cephalosporin, and macrolides) in Period 1 and Period 2. Error bars representing 95% confidence intervals and *p*-values were included.

Table 3 reports data concerning primary and secondary objectives of the study. Regarding the primary objective, systemic antibiotic therapy at discharge was recommended for 2333 (47.8%) patients of the total population; 1236 (56.7%) and 1096 (40.6%) in Periods 1 and 2, respectively (*p* < 0.001). Figure 1 and Figure 2 show differences in antibiotic prescriptions between the two study periods.

A statistically significant reduction was observed in the utilization of other diagnostic tests, with laboratory blood tests decreasing by 20.2% and CXRs by 37% (*p* < 0.001). Median (IQR) values of CRP at laboratory blood test were 1.97 (0.77–5.45) mg/dL in the total population, 1.99 (0.72–5.21) mg/dL in Period 1, and 1.94 (0.84–5.58) mg/dL in Period 2 (*p* > 0.05). Even the post hoc analysis of discharge types from PED revealed a statistically significant increase in the proportion of patients discharged home (*p* < 0.001) and a reduction in those admitted to the SSO (*p* = 0.002), during Period 2.

Table 4 describes the data related to the diagnostic assessments (POCTs and multiplex PCR tests) carried out in PED. In Period 2, there was a 76.5% and a 56.6% increase in the utilization of the GAS-RAD and of the RSV-RAD tests, respectively (*p* < 0.001). GAS-RAD tests yielded positive results in 173 (69.2%) and 302 (55.1%) cases in Periods 1 and 2, respectively. In particular, in patients with a diagnosis of URTI, this test was positive in 176 (25.7%) and 302 (49.4%) cases in Periods 1 and 2, respectively.

The RSV-RAD test yielded positive results in 36 (31.3%) and 125 (55.8%) cases in Periods 1 and 2, respectively.

In Period 2, COMBO tests resulted positive for Influenza A in 303 patients (38%) and for Influenza B in 6 patients (0.8%). Median (IQR) values of CRP-POC were 1.61 (0.5–3.5) mg/dL.

In Table S1, we compared subgroups of patients in Period 2 who did and did not use the new POCTs (PCR-POC and COMBO tests). There was a statistically significant difference between the two periods for the following variables: age (*p* < 0.001), weight (*p* < 0.001), heart rate (*p* < 0.001), body temperature (*p* < 0.001), fever duration (*p* < 0.001), body temperature ≥ 38 °C (*p* < 0.001), comorbidity (*p* = 0.005), type of comorbidity (*p* = 0.006) and discharge diagnosis (*p* < 0.001). Regarding diagnosis, the post hoc analysis of diagnoses at discharge showed that we performed fewer POCTs on patients with croup (*p* < 0.001) and more POCTs on patients with bronchiolitis (*p* < 0.001). No statistically significant differences were observed for URTI, bronchitis, and pneumonia. All effect sizes are small.

The comparison between the variables of the two periods in Table 2, Table 3 and Table 4 showed that all effect sizes are small.

Univariate and multivariate logistic regression analyses are reported in Table 5.

## 4. Discussion

The present study investigates the impact of POCTs and multiplex-PCR tests on the routine clinical practice of children with febrile ARIs in a PED of a tertiary care university hospital.

The main finding is that prescriptions of systemic antibiotic therapy at PED discharge in children admitted for ARIs significantly decreased in Period 2, when the tests were implemented. Furthermore, it is important to note that in our subgroup of patients with URTIs, almost half of the antibiotic prescriptions are likely due to GAS-RAD positivity. These data suggest that, in PED at the IRCCS Azienda Ospedaliero-Universitaria, there is an improvement in the appropriateness of antibiotic prescriptions and adherence to national clinical guidelines [7,8,25,26]. Antibiotic prescription rates in the pediatric population in 2016–2017 were comparable to those seen in 2023–2024, according to the Emilia Romagna Region’s 2024 report on antibiotic use and antimicrobial resistance in children. Prescriptions actually decreased during the SARS-CoV-2 pandemic before rising once more after 2021 due to the removal of containment measures that had prevented the spread of infectious pathogens in the area [27].

Amoxicillin remained the most frequently prescribed antibiotic in both periods, with an improvement in the ratio of narrow-spectrum to broad-spectrum antibiotics in Period 2. Concurrently, the median duration of antibiotic therapy prescription for uncomplicated ARIs remains higher than that currently suggested in the literature. However, the assessment of prescriptive appropriateness lies beyond the scope of this study. This necessitates the implementation of targeted improvement measures that encompass multiple factors, which cannot be addressed only by a single resource such as POCTs [28,29].

A number of studies have been conducted with the objective of identifying the factors that influence antibiotic prescription in paediatric patients [30,31,32]. The present study shows a positive correlation between age and the likelihood of antibiotic prescription. Although younger children experience a higher incidence of viral respiratory infections, which generally do not require antibiotic therapy [33], several studies have reported higher rates of antibiotic prescribing in this age group [34,35]. A European study among febrile children referred to the PED reported findings consistent with our results, indicating that a longer duration of fever and older age are associated with an increased likelihood of antibiotic prescription [31]. In our cohort, the presence of fever, defined as a body temperature of at least 38 °C, significantly increased the likelihood of antibiotic use. Moreover, each additional day of fever prior to PED presentation was associated with a further increase in this probability. Contrary to some previous reports, comorbidities were not significantly associated with antibiotic prescribing [36]. This finding aligns with the study by Covino et al., which identified older age, performance of CXRs, fever > 40 °C, and overall poor clinical condition as significant predictors of antibiotic use, rather than the presence of comorbidities [32].

About the diagnosis, patients with URTI, croup, bronchitis, and bronchiolitis were found to be significantly less likely to receive antibiotic therapy than those with pneumonia. This finding is in accordance with the literature and current guidelines [8,25,26].

Interestingly, the likelihood of receiving an antibiotic prescription was lower when certain diagnostic tools were used, including laboratory blood tests, multiplex PCR testing, and the newly introduced POCTs during Period 2 (CRP-POC and COMBO test). In particular, our analysis considered whether diagnostic tests were performed, rather than the results obtained from them. However, it is important to note that median CRP values are not elevated and are comparable in the two periods. At the same time, supporting the high prevalence of ARIs in children [3,4], the positivity rates of the COMBO test and multiplex PCR test are relatively high.

In recent years, several studies have investigated the usefulness of POCTs and multiplex PCR tests in reducing antibiotic prescriptions. A 2022 systematic review and meta-analysis of 57 paediatric studies on respiratory panel film arrays and rapid Influenza tests found that patients who tested positive for Influenza had a significantly lower rate of antibiotic prescriptions than those who tested negative [15].

The utility of multiplex PCR tests has been the subject of conflicting results in the extant literature [37]. It is important to note that randomized clinical trials by Mattila et al. and Rao et al. found no significant reduction in antibiotic prescription rates among pediatric patients who underwent virological diagnostic testing compared to control groups [23,38]. Conversely, other studies advocated for the utilization and implementation of such microbiological tests, underscoring their significance in establishing etiological diagnosis and their capacity to enhance appropriate antibiotic prescriptions [11,39].

In the context of CRP-POC tests, recent studies have sought to elucidate the role of biomarkers (CRP and procalcitonin) in paediatric ARIs. Even though their role in distinguishing bacterial from viral etiology is not yet fully established, biomarkers are widely utilized by clinicians to support diagnostic decision-making. Notably, the implementation of CRP-POC tests in the PED has increased in recent years, reflecting their potential utility in guiding antibiotic prescribing [8,11,40].

Indeed, in paediatric patients, the use of CRP-POC tests with a predefined cut-off value as a guiding threshold seems to be associated with a reduction in antibiotic prescriptions.

This association was highlighted in a recent systematic review encompassing 13 studies and a total of 9844 subjects, which evaluated the impact of CRP-POC testing on antibiotic prescribing in adults and children with upper and lower respiratory tract infections [41].

Furthermore, a 2022 Cochrane systematic review examining the use of POCTs in respiratory infections demonstrated that CRP-POC testing, when used alongside clinical assessment and patient history, led to decreased antibiotic prescriptions both immediately and within 28 days of follow-up [21].

During Period 2, the use of CXRs and laboratory blood tests (excluding CRP-POC) showed a statistically significant reduction, while the utilization of existing POCTs increased, alongside the introduction of novel POCTs and the implementation of multiplex PCR tests. In accordance with the findings of the present study, other authors have indicated a decline in the utilization of blood tests and CXRs subsequent to the implementation of virological tests [22,42]. In line with the observations of Bellini et al., the frequency of laboratory blood tests was found to be significantly lower than in children who tested positive for respiratory viruses using POCTs compared to those who tested negative (38.2% vs. 51.7%) [43].

Nevertheless, in Period 2, an increase in blood test utilization was observed when CRP-POC tests were included. This likely reflects the ability of CRP-POC testing to reduce diagnostic uncertainty and provide rapid results, thereby promoting more frequent use. Supporting this, a study conducted in England, based on interviews with 24 healthcare professionals, identified key factors influencing POCTs’ utilization: minimal invasiveness, ease of use, rapid result availability, and the capacity to facilitate more targeted antibiotic therapy [44].

During Period 2, there was an increase in the number of patients discharged home and a decrease in the number of patients admitted to the SSO. These changes may be associated with the greater etiological certainty provided by diagnostic information obtained from POCTs [32]. Concurrently, the LOS in the PED exhibited a slight increase, with a median rise of 16 min, though this change is not deemed to be clinically significant. This could be attributed to several factors that are not easily identifiable, including the additional time required to perform POCT and multiplex PCR tests, a higher number of patients in the PED, or greater case complexity. [23]. This is in contrast to other findings in which the use of POCTs is associated with a reduction in LOS [15].

A marginal rise has been observed in the utilization of PED access for cases of febrile ARIs in Period 2. It is important to note that following the considerable decrease in PED visits that was observed during the period of the SARS-CoV-2 pandemic, the 2023–2024 epidemic season has returned to pre-pandemic levels [28,45].

With regard to diagnostic investigations, Period 2 witnessed a substantial rise in the utilization of GAS-RAD and RSV-RAD tests. Indeed, in the post-pandemic era of SARS-CoV-2, there has been a heightened focus on microbiological diagnostic tests, accompanied by the integration of POCTs in PEDs and the increased utilization of existing diagnostic tools [15].

Furthermore, approximately one-third of the study population underwent rapid COMBO testing during Period 2. The positivity for Influenza A was found to be significantly higher in comparison to Influenza B, thereby confirming data from the RespiVirNet integrated respiratory virus surveillance in Italy for the 2023–2024 season. This surveillance demonstrated a clear predominance of Influenza A virus during that season [29].

The multiplex PCR test was performed in approximately one-third of patients, with higher positivity rates than those reported in other studies [33]. Specifically, the most prevalent virus identified in Period 2 was Influenza A, followed by Rhinovirus, Respiratory Syncytial virus, and Adenovirus. With regard to the 2023–2024 season, there is a slight discrepancy between our data and the RespiVirNet integrated respiratory virus surveillance in Italy. In Italy, the most prevalent virus is Influenza A, followed by SARS-CoV-2, Respiratory Syncytial virus, and Rhinovirus. However, it is important to note that the time period is slightly different and also includes data on adult patients and SARS-CoV-2 infections [29].

Over the years, the Microbiology Unit has moved from using direct immunofluorescence assays to detect respiratory virus antigens in nasopharyngeal aspirates to multiplex PCR testing, which was also validated for use on nasopharyngeal swabs. The use of nasopharyngeal swabs rather than aspirates is less invasive for the patient and quicker to perform for healthcare personnel.

The strengths of this study include its substantial sample size, the collection of a plethora of variables and data, and the comparison with a pre-implementation period. The primary limitations of the study arise from its retrospective and monocenter design, and from the potential bias related to the study design. Another limitation is the potential impact of confounding factors, such as changes in pathogen circulation and viral epidemiology over time, which may influence prescribing patterns. Additionally, shifts in clinical practice, including updates to pediatric guidelines and increased awareness of antimicrobial resistance, could also affect antibiotic prescribing trends. The present study lends support to the notion that rapid diagnostics and multiplex PCR tests can function as tools in the field of PED, given that they could have the capacity to reduce diagnostic uncertainty, decrease the potential inappropriate prescription of antibiotics, and reduce the utilization of conventional diagnostic tests (e.g., laboratory blood tests and CXRs).

## 5. Conclusions

In recent years, there has been significant development in the field of diagnostic stewardship, alongside the integration of POCTs in PEDs. These tests are valuable for differentiating between bacterial and viral infections and determining the etiology of ARIs. In addition, they could assist clinicians in selecting the most appropriate therapeutic approach. In the context of the PED, the implementation of POCTs and the multiplex PCR test could be a valuable tool for reducing antibiotic prescriptions, as well as the use of laboratory blood tests and CXRs in patients presenting with febrile ARIs. This has not been without its advantages for children, the community, and the healthcare system.

Furthermore, these data facilitate the development of protocols in PED to standardize the use of POCTs and multiplex PCR testing. Moreover, conducting a cost–benefit analysis would be advantageous in order to ascertain whether the implementation of new diagnostic tests would result in a favorable impact on healthcare costs.

## Figures and Tables

**Table 1:** Type of tests present in Period 1 and Period 2 in the Pediatric Emergency Department of IRCCS Azienda Ospedaliero-Universitaria, and the type of tests present in Period 1 and Period 2. GAS-RAD = Group A Streptococcus rapid antigen detection; RSV-RAD = Respiratory Syncytial virus rapid antigen detection; DFAs = direct immunofluorescence assays; PCR = polymerase chain reaction; COMBO = rapid multiple antigen test for SARS-CoV-2, Influenza A, and Influenza B; CPR-POC = C-Reactive protein point-of-care.

	Period 1	Period 2
GAS-RAD test	X	X
RSV-RAD test	X	X
DFAs	X	
SARS-CoV-2 antigenic test		X
Multiplex PCR test		X
CRP-POC		X
COMBO test		X

**Table 2:** Characteristics of the study cohort compared by Period 1 and Period 2. SD = standard deviation, IQR = interquartile range, SpO2 = saturation of peripheral oxygen, URTI = upper respiratory tract infection, n.s. = not significant.

	Total Population n = 4882	Period 1 n = 2181	Period 2 n = 2701	*p*
Female, n (%)	2206 (45.2%)	975 (44.7%)	1231 (45.6%)	n.s.
Age [months], Median (IQR)	32 (15–62)	28 (14–53)	37 (16–70)	<0.001
Weight [kg], Median (IQR)	14 (10–20)	13 (10–18)	14 (10.5–20)	<0.001
Heart Rate [bpm], Mean (SD)	137 (23.34)	139 (23.52)	135 (23.02)	<0.001
SpO2 [%], Median (IQR)	98 (97–99)	98 (97–99)	98 (97–99)	<0.001
Body Temperature [°C], Mean (SD)	37.7 (1.1)	37.8 (1.1)	37.6 (1.1)	<0.001
Fever duration [days], Median (IQR)	2 (1–3)	2 (1–3)	2 (1–4)	<0.001
Body temperature ≥ 38°C, n (%)	4216 (86.4%)	1856 (85.1%)	2360 (87.4%)	0.024
Antibiotic prior to PED, n (%)				n.s.
-Amoxicillin	348 (44%)	150 (40.9%)	198 (46.8%)	
-Amoxicillin-clavulanic acid	227 (28.7%)	106 (28.9%)	121 (28.6%)	
-Cephalosporin	106 (13.5%)	54 (14.7%)	52 (12.3%)	
-Macrolides	109 (13.8%)	57 (15.5%)	52 (12.3%)	
Type of comorbidity, n (%)				<0.001
-Heart disease	56 (1.2%)	25 (1.2%)	31 (1.2%)	
-Neurological disease	51 (1.0%)	22 (1%)	29 (1.1%)	
-Metabolic disease	14 (0.3%)	7 (0.3%)	7 (0.3%)	
-Genetic syndrome	44 (1%)	8 (0.4%)	36 (1.3%)	
-Kidney disease	36 (0.7%)	19 (0.9%)	17 (0.6%)	
-Immunodeficiency	5 (0.1%)	2 (0.1%)	3 (0.1%)	
-Oncological disease	25 (0.5%)	12 (0.6%)	13 (0.5%)	
-Autoimmune disease	4 (0.1%)	1 (0.1%)	3 (0.1%)	
-Lung disease	150 (3.1%)	43 (2%)	107 (4%)	
-Prematurity	54 (1.1%)	19 (0.9%)	35 (1.3%)	
-Endocrine disease	10 (0.2%)	6 (0.3%)	4 (0.2%)	
-Febrile seizure	116 (2.4%)	60 (2.8%)	56 (2.1%)	
Discharge diagnosis, n (%)				<0.001
-URTI	2986 (61.2%)	1253 (57.5%)	1733 (64.2%)	
-Croup	243 (5%)	136 (6.2%)	107 (4%)	
-Bronchitis	940 (19.2%)	469 (21.5%)	471 (17.4%)	
-Bronchiolitis	263 (5.4%)	120 (5.5%)	142 (5.3%)	
-Pneumonia	450 (9.2%)	203 (9.3%)	247 (9.1%)	

**Table 3:** Antibiotic prescription, type of antibiotic prescription, execution of blood test and chest radiographs, access duration, and type of discharge between Period 1 and Period 2. IQR = interquartile range, URTI = upper respiratory tract infection, SSO = short-stay observation, LOS = length of stay; CXR = chest X-ray. ° New prescription refers to starting antibiotic therapy in untreated patients. * The percentage is relative to the respective diagnosis of discharge.

	Total Population n = 4882	Period 1 n = 2181	Period 2 n = 2701	*p*
Antibiotic prescription, n (%)				<0.001
-new prescription °	1909 (39.1%)	1053 (48.3%)	855 (31.7%)	
-continue antibiotic	424 (8.7%)	183 (8.4%)	241 (8.9%)	
Antibiotic prescription by diagnosis, n (% *):				<0.001
-URTI	1286 (43.1%)	674 (53.8%)	612 (35.3%)	
-Croup	59 (24.3%)	45 (33.1%)	14 (13.1%)	
-Bronchitis	586 (62.3%)	329 (70.1%)	257 (54.6%)	
-Bronchiolitis	33 (12.5%)	22 (18.3%)	11 (7.7%)	
-Pneumonia	369 (82%)	166 (81.8%)	203 (82.2%)	
Type of systemic antibiotic, n (%)				<0.001
-Amoxicillin	1082 (46.4%)	532 (43%)	550 (50.2%)	
-Amoxicillin-clavulanic acid	659 (28.3%)	390 (31.6%)	269 (24.5%)	
-Cephalosporin	302 (12.9%)	174 (14.1%)	128 (11.7%)	
-Macrolides	289 (12.4%)	140 (11.3%)	149 (13.6%)	
Length of therapy [days], Median (IQR)	7 (6–7)	7 (7–7)	7 (6–7)	0.004
Laboratory blood tests performance, n (%)	835 (17.1%)	420 (19.3%)	415 (15.4%)	<0.001
CXRs performance, n (%)	807 (16.5%)	454 (20.8%)	353 (13.1%)	<0.001
LOS [minutes], Median (IQR)	107 (54–193)	97 (45–192)	113 (60–194)	<0.001
Type of discharge, n (%)				0.001
-Home	4235 (86.8%)	1845 (84.6%)	2390 (88.5%)	
-SSO	289 (5.9%)	159 (7.3%)	130 (4.8%)	
-Hospitalization	358 (7.3%)	177 (8.1%)	181 (6.7%)	

**Table 4:** Rapid diagnostic tests and multiplex PCR tests in PSP between Period 1 and Period 2. CRP-POC = C-Reactive Protein Point-of-Care, GAS-RAD = Group A Streptococcus rapid antigenic detection; RSV-RAD = Respiratory Syncytial virus rapid antigenic detection; PCR = Polymerase Chain Reaction; COMBO test = rapid multiple antigen test for SARS-CoV-2, Influenza A and Influenza B.

	Total Population n = 4882	Period 1 n = 2181	Period 2 n = 2701	*p*
CRP-POC, n (%)			447 (16.5%)	
GAS-RAD, n (%)	798 (16.3%)	250 (11.5%)	548 (20.3%)	<0.001
RSV-RAD, n (%)	339 (6.9%)	115 (5.3%)	224 (8.3%)	<0.001
Rapid COMBO test, n (%)			796 (29.5%)	
Multiplex PCR test, n (%)			783 (29%)	
-Influenza A positive, n (%)			269(34.4%)	
-Influenza B positive, n (%)			6 (0.8%)	
-Parainfluenza positive, n (%)			31 (4%)	
-Rhinovirus positive, n (%)			160 (20.4%)	
-Metapneumovirus positive, n (%)			43 (5.5%)	
-Adenovirus positive, n (%)			81 (10.3%)	
-Respiratory Syncytial virus positive, n (%)			149 (19%)	

**Table 5:** Univariate and multivariate logistic regression analysis considering antibiotic prescription as the dependent variable. OR = Odds Rate, CI = confidence interval, LOS = length of stay; CXR = Chest X-ray; CRP = C-reactive Protein; PCR = Polymerase Chain Reaction; URTI = upper respiratory tract infection; COMBO test = rapid multiple antigen test for SARS-CoV-2, Influenza A and Influenza B; POCTs = point-of-care tests; n.s. = not significant.

	Univariate			Multivariate		
	OR	IC 95%	*p*	OR	IC 95%	*p*
Period 1	1.91	1.71–2.14	<0.001	2.01	1.73–2.33	<0.001
LOS	1	1.00–1.00	n.s.			
Age (months)	1.01	1.01–1.01	<0.001	1.01	1.01–1.01	<0.001
Sex	1.05	0.94–1.18	n.s.			
Days of fever	1.21	1.18–1.25	<0.001	1.19	1.16–1.24	<0.001
Body temperature ≥ 38 °C	2.2	1.85–2.63	<0.001	2.14	1.76–2.62	<0.001
Comorbidity	0.9	0.76–1.08	n.s.			
Laboratory blood tests	0.88	0.76–1.02	0.093	0.66	0.56–0.79	<0.001
CXR	2.47	2.11–2.90	<0.001	1.03	0.83–1.28	n.s.
POCTs (CRP + COMBO test)	0.55	0.48–0.63	<0.001	0.76	0.63–0.92	0.004
Multiplex PCR test	0.5	0.43–0.59	<0.001	0.66	0.54–0.81	<0.001
Diagnosis (ref pneumonia):	1.29	1.23–1.34	<0.001			
-URTI	0.17	0.12–0.21	<0.001	0.18	0.13–0.24	<0.001
-croup	0.07	0.05–0.1	<0.001	0.07	0.05–0.11	<0.001
-bronchitis	0.36	0.27–0.48	<0.001	0.41	0.3–0.55	<0.001
-bronchiolitis	0.03	0.02–0.05	<0.001	0.05	0.03–0.09	<0.001

## Data Availability

The data presented in the study are available from the corresponding author upon reasonable request.

## Supplementary Materials

The following supporting information can be downloaded at https://www.mdpi.com/article/10.3390/pathogens14121284/s1. Table S1: Comparison between the subgroup in period 2 that received new POCTs (PCR-POC and COMBO test) and the subgroup in period 2 that did not receive POCTs. POCT = point-of-care test, SD = standard deviation, IQR = interquartile range, SpO2 = saturation of peripheral oxygen, URTI = upper respiratory tract infection, n.s. = non significant.

## Abbreviations

The following abbreviations are used in this manuscript:

ARIs	Acute Respiratory Infections
PED	Pediatric Emergency Department
URTIs	Upper Respiratory Tract Infections
POCTs	Point-of-Care Tests
GAS-RAD	Group A Streptococcus rapid antigen detection
RSV-RAD	Respiratory Syncytial Virus rapid antigen detection
DFAs	Direct Immunofluorescence Assays
PCR	Polymerase Chain Reaction
CRP-POC	C-Reactive Protein Point-of-Care
LOS	Length Of Stay
SSO	Short-Stay Observation
CXRs	Chest X-rays
CRREM	Regional Reference Center for Microbiological Emergencies
SD	Standard Deviation
IQR	Interquartile Range
SpO2	Saturation of Peripheral Oxygen
CRP	C-Reactive Protein
OR	Odds Rate
CI	Confidence Interval
