# Supplementary material for: The Use of Point-of-Care Tests and Multiplex PCR Tests in the Pediatric Emergency Department Reduces Antibiotic Prescription in Patients with Febrile Acute Respiratory Infections
Source: Pathogens. 2025 Dec 13;14(12):1284. doi: 10.3390/pathogens14121284 (PMC12735915; doi:10.3390/pathogens14121284)
Supplement: Supplementary file 1 [file pathogens-14-01284-s001.zip › pathogens-3995949-supplementary.pdf]

pathogens

MDPI

Table S1. Comparison between the subgroup in period 2 that received new POCTs (PCR-POC and COMBO test) and the subgroup in period 2 that did not receive POCTs. POCT = point-of-care test, SD = standard deviation, IQR = interquartile range, SpO2 = saturation of peripheral oxygen, URTI = upper respiratory tract infection, n.s. = non significant

	**NO POCT n= 1647**	**POCT n= 1054**	**p**
Female, n (%)	743 (45.1%)	488 (46.3%)	n.s.
Age [months], Median (IQR	41 (19-75)	30 (13-63)	<0.001
Weight [kg], Median (IQR)	15 (11-22)	13 (10-19)	<0.001
Heart Rate [bpm], Mean (SD)	133 (22.4)	138 (23.6)	<0.001
SpO2 [%], Median (IQR)	98 (97-99)	98 (97-99)	n.s.
Body Temperature [°C], Mean (SD)	37.5 (1.1)	37.7 (1.2)	<0.001
Fever duration [days], Median (IQR)	2 (1-3)	2 (1-4)	<.001
Body temperature ≥ 38°C, n (%)	1408 (85.49%)	952 (90.32%)	<0.001
Antibiotic prior to PED, n (%)			n.s
- Amoxicillin	120 (51.1%)	78 (41.5%)	
- Amoxicillin-clavulanic acid	61 (26%)	60 (31.9%)	
- Cephalosporin	29 (12.3%)	23 (12.2%)	
- Macrolides	25 (10.6%)	27 (14.4%)	
Comorbidity, n (%)	183 (11.2%)	157 (14.9%)	0.005
Type of comorbidity, n (%)			0.006
- Heart disease	16 (8.7%)	15 (9.6%)	
- Neurological disease	13 (7.1%)	16 (10.2%)	
- Metabolic disease	7 (3.8%)	0 (0%)	
- Genetic syndrome	14 (7.6%)	22 (14%)	
- Kidney disease	10 (5.4%)	7 (4.5%)	
- Immunedeficiency	1 (0.5%)	2 (1.3%)	
- Oncological disease	4 (2.2%)	9 (5.7%)	
- Autoimmune disease	2 (1.1%)	1 (0.6%)	
- Lung disease	69 (37.5%)	38 (24.2%)	
- Prematurity	23 (1.5%)	12 (7.6%)	
- Endocrine disease	3 (1.6%)	1 (0.6%)	
- Febrile seizure	22 (12%)	34 (21.7%)	
Discharge diagnosis, n (%)			<0.001
- URTI	1074 (65.2%)	659 (62.5%)	
- Croup	88 (5.3%)	19 (1.8%)	

- Bronchitis	302 (18.3%)	169 (16%)
- Bronchiolitis	48 (2.9%)	95 (9%)
- Pneumonia	135 (8.2%)	112 (10.6%)
